# Cancer patients in Palliative Care: occurrences related to venipuncture and hypodermoclysis

**DOI:** 10.1590/1518-8345.5825.3624

**Published:** 2022-08-12

**Authors:** Fabiana Bolela, Roberta de Lima, Ana Carolina de Souza, Michele Rocha Moreira, Ana Julia de Oliveira Lago, Giovana Paula Rezende Simino, Jakeline Silva de Araújo

**Affiliations:** 1 Universidade de São Paulo, Escola de Enfermagem de Ribeirão Preto, Centro Colaborador da OPAS/OMS para o Desenvolvimento da Pesquisa em Enfermagem, Ribeirão Preto, SP, Brasil.; 2 Instituto Nacional de Câncer José Alencar Gomes da Silva, Hospital do Câncer IV, Rio de Janeiro, RJ, Brasil.; 3 Universidade Federal de Minas Gerais, Escola de Enfermagem, Belo Horizonte, MG, Brasil.

**Keywords:** Palliative Care, Hypodermoclysis, Subcutaneous Infusions, Peripheral Catheterization, Medical Oncology, Nursing, Cuidados Paliativos, Hipodermóclise, Infusões Subcutâneas, Cateterismo Periférico, Oncologia, Enfermagem, Cuidados Paliativos, Hipodermoclisis, Infusiones Subcutáneas, Cateterismo Periférico, Oncología Médica, Enfermería

## Abstract

**Objective:**

to identify the occurrences related to peripheral venipuncture and hypodermoclysis among patients hospitalized in a general hospital and in an exclusive hospital institution for the care of patients in palliative cancer care.

**Method:**

an observational, descriptive and multicenter study. The consecutive and non-probabilistic sample consisted of 160 cancer patients hospitalized in Palliative Care. The outcome variable corresponded to the occurrences and complications related to each type of puncture. A questionnaire containing the sociodemographic and clinical variables and a structured script for monitoring and daily evaluation of the puncture were used. Descriptive statistics were employed for data analysis.

**Results:**

the occurrences related to venipuncture at a general hospital were blood soiling at catheter insertion (17.4%) and expired use period (15.8%), while at a specific service for the care of patients under palliative care they were expired use period (32%) followed by infiltration (18.9%). As for hypodermoclysis, there were two subcutaneous punctures with phlogistic signs (1.0%) at the general hospital and a hematoma at the catheter insertion site (0.5%). At the specific service for the care of patients under palliative care there were three subcutaneous punctures with phlogistic signs (5.7%).

**Conclusion:**

the number of occurrences related to peripheral venipuncture was higher than those related to hypodermoclysis.

## Resumo

**Objetivo:**

identificar as ocorrências relacionadas à punção venosa periférica e à hipodermóclise entre pacientes internados em um hospital geral e em um hospital exclusivo de assistência a pacientes em cuidados paliativos oncológicos.

**Método:**

estudo observacional, descritivo e multicêntrico. A amostra do tipo consecutiva e não probabilística foi constituída por 160 pacientes oncológicos internados sob cuidados paliativos. A variável desfecho correspondeu às ocorrências e complicações relacionadas a cada tipo de punção. Utilizou-se um questionário contendo as variáveis sociodemográficas e clínicas e um roteiro estruturado para acompanhamento e avaliação diária da punção. Foram utilizadas estatísticas descritivas para a análise dos dados.

**Resultados:**

as ocorrências relacionadas à punção venosa no hospital geral foram sujidade de sangue na inserção do cateter (17,4 %) e prazo de uso expirado (15,8%), enquanto no serviço específico para atendimento a pacientes sob cuidados paliativos foram prazo de uso expirado (32%) seguido de infiltração (18,9%). Quanto à hipodermóclise, foram duas punções subcutâneas com sinais flogísticos (1,0%) no hospital geral e um hematoma no local de inserção do cateter (0,5%). No serviço específico para atendimento a pacientes sob cuidados paliativos foram três punções subcutâneas com sinais flogísticos (5,7%).

**Conclusão:**

as ocorrências relacionadas à punção venosa periférica foram superiores às relacionadas à hipodermóclise.

## Resumen

**Objetivo:**

identificar los eventos relacionados con la venopunción periférica y la hipodermoclisis en pacientes hospitalizados en un hospital general y en un hospital que atiende exclusivamente a pacientes en cuidados paliativos oncológicos.

**Método:**

estudio observacional, descriptivo y multicéntrico. La muestra consecutiva y no probabilística estuvo compuesta por 160 pacientes oncológicos hospitalizados que reciben cuidados paliativos. La variable resultado correspondió a los eventos y complicaciones relacionados con cada tipo de punción. Se utilizó un cuestionario con variables sociodemográficas y clínicas y una guía estructurada para el seguimiento diario y la evaluación de la punción. Se utilizó estadística descriptiva para el análisis de datos.

**Resultados:**

los eventos relacionados con la venopunción en un hospital general fueron contaminación de sangre en la inserción del catéter (17,4%) y catéter vencido (15,8%), mientras que en un hospital específico que atiende exclusivamente a pacientes en cuidados paliativos oncológicos fueron catéter vencido (32%) seguido de infiltración (18,9%). En cuanto a la hipodermoclisis, hubo dos punciones subcutáneas con signos flogísticos (1,0%) en hospital general y un hematoma en el lugar de inserción del catéter (0,5%). En el hospital en un hospital que atiende exclusivamente a pacientes en cuidados paliativos oncológicos hubo tres punciones subcutáneas con signos flogísticos (5,7%).

**Conclusión:**

los eventos relacionados con la venopunción periférica fueron mayores que los relacionados con la hipodermoclisis.

**Highlights**

(1) There were more complications related to venipuncture than to hypodermoclysis.

(2) Hypodermoclysis offers greater patient safety.

(3) It is recommended to train the professionals on the use of hypodermoclysis.

(4) Teaching the hypodermoclysis practice must be encouraged in universities.

(5) The study may contribute to greater adherence to hypodermoclysis.

## Introduction

Palliative Care (PC) is mainly offered to people who are at the end of life without age restriction, who face intense health-related distress due to serious diseases. The objective of PC is to offer active holistic care and improve the quality of life of patients, their families and their caregivers^1^.

Hospitalization of patients in PC is recurrent due to the need to control the signs and symptoms they present, required by the complications related to diagnosis and evolution of the disease. In such situations, it is necessary to obtain an access route for parenteral drug therapy^2^.

Thus, some technological options in the health area and in the development of PC therapy have been adopted, such as techniques and options for the administration of fluids and medications. Alternative routes need to be considered for this purpose, as patients in PC usually present difficulties and/or impossibility of oral administration of medications in the face of the symptoms presented^3^ ^-^ ^4^.

The use of peripheral venous catheters (PVCs) for intravenous administration of medications and solutions has become an indispensable resource for care in the hospital environment^5^. However, a number of studies have documented high incidence of peripheral vascular trauma during PVC use, in addition to other complications, the most frequent being phlebitis, infection at the catheter insertion site, bacteremia and sepsis, reinforcing that use of such devices is not free from risks of complications^5^ ^-^ ^8^.

Given the above, considering the profile of patients undergoing PC in whom fragility of the venous network often hinders or prevents puncture of a peripheral vein, it is necessary to consider other possible routes, one of which is puncturing the subcutaneous route, or hypodermoclysis.

The term hypodermoclysis refers to the subcutaneous (SC) infusion of isotonic fluids and/or medications^9^. It is a simpler procedure than peripheral venipuncture, safe and without serious complications. However, the technique is still poorly disseminated and used in the clinical practice^2^ ^,^ ^10^.

Complications related to using the SC route are rare when proper use of the puncture technique, dilution and infusion of medications is adopted^11^ ^-^ ^12^. In addition to that, use of the SC route is less costly and less invasive than using the intravenous route^11^.

However, it is worth noting that hypodermoclysis has different indications and its use should be carefully evaluated, taking into account the characteristics of the patient and the medications prescribed, among others^12^ ^-^ ^13^.

In the national scenario, publications on the complications related to hypodermoclysis when compared to peripheral venipuncture are restricted to review studies^4^ ^,^ ^14^.

In view of the above, the objective of this study was to identify the occurrences related to punctures among patients hospitalized in a general hospital and in an exclusive hospital institution for the care of patients in palliative cancer care.

## Method

### Study design

An observational, descriptive and multicenter study. 

### Study locus

The study was conducted in two health services. One of the services included the medical clinic wards of the Ribeirão Preto State Hospital (*Hospital Estadual de Ribeirão Preto*, HERP), a public, secondary-level teaching hospital in the inland of the state of São Paulo. This service has a total of 50 beds, divided into two wards with 25 beds each, and has 10 exclusive beds for hospitalization of patients in Palliative Care. Patients admitted to this service are usually referred by emergency care units or general hospitals for the control of signs and symptoms or end-of-life care. In this service, the mean monthly hospitalization rate of patients in Palliative Care is approximately 17. 

The other health service was the IV Cancer Hospital (*Hospital do Câncer*IV, HC IV) - Palliative Care Unit of the José Alencar Gomes da Silva National Cancer Institute (*Instituto Nacional de Câncer*, INCA), located in the city of Rio de Janeiro. HC IV has a hospital structure for the comprehensive and active care of all patients with advanced cancer and no possibility of cure. The patients come from the Hospital Units of the INCA complex through the Outposts with a mean of 153 patients referred/month. After enrolling in HC IV, the patients are treated in one of the three follow-up modalities: hospitalization, home care or outpatient care. The hospitalization sector has 56 beds, divided into four floors for patients referred for the control of signs and symptoms, patients undergoing elective surgical procedures and patients in end-of-life care, and the monthly mean hospitalization rate is 170 patients. 

### Period

The recruitment period took place between January 2019 and February 2020.

### Population and sample

The sample was consecutive and non-probabilistic, consisting of 160 cancer patients hospitalized in Palliative Care. The potential participants were identified by a daily active search in the inpatient units. The eligibility criteria were verified at that moment. The potential participants were invited to the research, which involved daily observation of the puncture (venous or subcutaneous) and evaluation of the patients’ clinical conditions. In situations in which the patients did not present due clinical conditions to consent to participate in the study, their companions were responsible for doing so.

### Selection criteria

The inclusion criteria were as follows: cancer patients over 18 years old, of both genders, hospitalized in Palliative Care (ICD Z51.5 recorded in the medical record) and with the need for a puncture for parenteral drug therapy during hospitalization, regardless of their clinical condition and cognitive status. Those who were admitted to the inpatient units with a catheter already punctured in another service were excluded. 

### Instruments used to collect the information

For this study, questionnaires were used to obtain the sociodemographic and clinical variables, as well as a script for evaluation and daily follow-up of the puncture. To obtain the sociodemographic variables, the researcher prepared a questionnaire containing the following variables: date of the interview, date of birth (for later calculation of age), gender, marital status, schooling (in full years of formal studies), and main caregiver. 

To obtain the clinical variables, the researcher prepared a questionnaire containing the following variables: underlying oncological disease/medical diagnosis, presence of metastasis, type of puncture, purpose of peripheral venipuncture or hypodermoclysis (hydration, analgesia, antibiotic therapy, palliative sedation, control of other symptoms). The questionnaire was submitted to three PC specialists for them to assess adequacy of the content to the research objectives. 

A systematized script structured and prepared by the researcher was used for evaluation and daily monitoring of venipuncture and hypodermoclysis. The daily observation was performed by a nurse who works in the service participating in the study, two students attending the undergraduate Nursing course and the main researcher. To guide the observations, a training session was conducted by the researcher addressing the project objectives and the script items aiming at accuracy of the data collected. Divided into two parts, the script contains the following items: date of the puncture, type of puncture (venipuncture or hypodermoclysis), number of venipuncture attempts, subcutaneous puncture site, venipuncture site, type of catheter used and caliber. The second part refers to venipuncture and hypodermoclysis follow-up and contains the following items: presence of phlogistic signs at the catheter insertion site and other occurrences or complications with the puncture. The script was elaborated based on the main aspects involving venipuncture and subcutaneous punctures and their maintenance and was submitted to three PC specialists for them to assess adequacy of the content to the research objectives. 

### Data collection

Data collection was carried out through interviews with the patients or their guardians, consultation of the medical records, daily evaluation and monitoring of the punctures (venipuncture or subcutaneous), from the moment they were obtained until the moment when there was no more indication (suspension of parenteral medications, hospital discharge or death of the patient). 

So that it was possible to equate the puncture techniques and maintenance of venous or subcutaneous catheters, the Standard Operating Procedures of both services were compared and, observing equivalence or absence of significant differences between them, it was possible to initiate data collection, in order to eliminate biases related to the procedures performed in each service.

Following a predetermined schedule, the research team members took turns over the weeks for data collection, which occurred daily and always in the morning. The primary outcome involved observation of the puncture-related occurrences or complications. The occurrences or complications included the following: soiling at the catheter insertion site, catheter displacement, catheter obstruction, infiltration or leakage, phlebitis and inadequate fixation, among others, which could make catheter permanence unfeasible or constitute a risk for more severe complications.

There were no follow-up losses of patients during the data collection period.

### Data treatment and analysis

The data were structured in Microsoft Excel spreadsheets, undergoing double entry and a verification stage to minimize transcription errors. For the sociodemographic and clinical characterization, descriptive statistics were used in order to summarize the diverse information of interest. The qualitative variables were described in terms of absolute and percentage frequency, and the quantitative variables were described using central tendency (mean) and dispersion (standard deviation) measures.

Initial planning of the study provided for the comparison between the number of occurrences and complications related to punctures in each service where collection took place, covering more robust data analyses. However, the discrepancy between the number of participants in each service precluded such analyses.

### Ethical aspects

The study was approved by the Ethics Committee of the Ribeirão Preto College of Nursing under CAAE number 91320318.1.3002.5440 and by the Ethics Committee of the National Cancer Institute under CAAE number 91320318.1.3001.5274. The participants and the researcher signed the Free and Informed Consent Form (FICF).

## Results

The study included 160 cancer patients hospitalized in Palliative Care, 119 (74.4%) in HERP and 41 (25.6%) at INCA. Table 1 presents the participants’ sociodemographic data according to the hospitalization locus.

**Table 1:** Sociodemographic characteristics of the participants according to the hospitalization locus. Ribeirão Preto, SP, Brazil, 2020

Variables	HERP*			INCA^†^		
	n	%	Mean	n	%	Mean
**Gender**						
Male	57	47.9		20	48.8	
Female	62	52.1		21	51.2	
**Age**			67.4			60.8
**Age groups**						
18 - 59	26	21.9		22	53.6	
>60	93	78.1		19	46.4	
**Marital status**						
Single	17	14.3		09	21.9	
Married/Consensual union	63	52.9		22	53.7	
Separated/Divorced	18	15.1		04	9.8	
Widowed	21	17.7		06	14.6	
**Schooling (full years)**			5.4			7.6
**Main caregiver^‡^**						
Family member	111	94.1		37	94.9	
Not a family member	07	5.9		02	5.1	

*HERP =*Hospital Estadual de Ribeirão Preto*; ^†^INCA =*Instituto Nacional de Câncer*; ^‡^Missed values = HERP=1 (0.8%) and INCA=2 (4.9%).

The clinical characteristics of the study participants are shown in Table 2.

**Table 2:** Clinical characteristics of the patients according to the hospitalization locus. Ribeirão Preto, SP, Brazil, 2020

Variables	HERP*		INCA^†^	
	n	%	n	%
**Primary malignant neoplasm**				
Colorectal	15	12.6	02	4.9
Lung	15	12.6	02	4.9
Head and neck	13	10.9	06	14.6
Female breast	13	10.9	04	9.8
Esophagus	09	7.6	01	2.4
Prostate	07	5.9	03	7.3
CNS^‡^	07	5.9	02	4.9
Pancreas	06	5.0	01	2.4
**Metastasis**				
Lung	33	17.3	10	13.0
Liver	28	14.6	9	11.7
Bone	26	13.6	14	18.2
CNS^‡^	13	6.8	4	5.2
Lymph nodes	12	6.3	16	20.8
Pleura	6	3.1	2	2.6
Intestine	4	2.0	0	-
Peritoneum	3	1.6	6	7.8

*HERP =*Hospital Estadual de Ribeirão Preto;* ^†^INCA =*Instituto Nacional de Câncer;* ^‡^CNS = Central Nervous System

Table 3 presents the total and types of punctures observed at each hospitalization *locus*.

**Table 3:** Total number of punctures observed according to the hospitalization locus. Ribeirão Preto, SP, Brazil, 2020

Variables	HERP* (394)		INCA^†^ (97)	
	n	%	n	%
**Puncture types**				
Peripheral venous	342	86.8	72	74.2
Subcutaneous	52	13.2	25	25.8

*HERP =*Hospital Estadual de Ribeirão Preto*; ^†^INCA =*Instituto Nacional de Câncer*

In HERP, the main purpose of the punctures was antibiotic therapy (34.7%) followed by analgesia (34%), while at INCA it was analgesia (37.7%) followed by hydration (21.3%).

With regard to the occurrences related to hypodermoclysis, in HERP, two subcutaneous punctures were identified with phlogistic signs (1.0%) and a hematoma at the catheter insertion site (0.5%). At INCA, three subcutaneous punctures presented phlogistic signs (5.7%). The occurrences related to the venipunctures are presented in Table 4.

**Table 4:** Occurrences related to venipuncture according to the hospitalization locus. Ribeirão Preto, SP, Brazil, 2020

Variables	HERP*		INCA^†^	
	n	%	n	%
**Occurrence**				
Blood soiling at catheter insertion site	32	17.4	3	5.7
Catheter with expired use period	29	15.8	17	32.0
Pulled/Exteriorized catheter	26	14.1	3	5.7
Infiltration	24	13.0	10	18.9
Fixation does not allow viewing the catheter insertion site	18	9.8	7	13.2
Occlusion (obstructed catheter)	12	6.5	2	3.8
Hematoma	9	4.9	3	5.7
Phlebitis - Grade 1	7	3.8	1	1.9
Partially loose dressing	6	3.3	0	-
Local pain	5	2.7	0	-
Phlebitis - Grade 2	5	2.7	0	-
Phlebitis - Grade 3	2	1.0	0	-
Maceration of the catheter insertion hole (hole widening)	2	1.0	0	-
Bent catheter	1	0.5	1	1.9
Phlebitis - Grade 4	2	1.0	0	-
Leakage at catheter insertion site	1	0.5	1	1.9

*HERP =*Hospital Estadual de Ribeirão Preto*; ^†^INCA =*Instituto Nacional de Câncer*

## Discussion

In this study, the number of female participants in both hospitalization loci was higher, corroborating other national and international studies^15^ ^-^ ^21^. Both in Brazil and in other developing countries, there is predominance of cancer in the female population, which is related to the high detection rates of gender-specific neoplasms such as cervical cancer^22^. 

Predominance of aged patients was observed in the current study. In a study that aimed at characterizing the patients evaluated by the Palliative Care service of a University Hospital in the Brazilian Southeast region, aged patients predominated in the population of interest^23^, as is also the case in some international studies^17^ ^-^ ^18^. A survey that aimed at evaluating the quality of life of cancer patients in Palliative Care obtained younger participants in its study, diverging from the findings of the current research^20^.

The advanced age of the patients participating in the study reinforces the demographic estimates that indicate the increase in life expectancy and, consequently, population aging. Thus, chronic-degenerative diseases should be considered priorities in health care^24^.

As in the current study, the authors identified that the most frequent marital status among the participants was married (52%)^23^. Another study that aimed at evaluating the health-related quality of life of cancer patients in Palliative Care and its association with sociodemographic and clinical aspects also obtained greater participation of married patients or in a consensual union^16^. A number of studies suggest a higher level of social support among patients with the presence of a caregiver. With the diagnosis of a chronic disease, feelings of threat to life, uncertainties and stress emerge and affect patients and families. Thus, the role of family members becomes paramount, favoring psychological adjustment as well as helping to manage the symptoms caused by the disease^25^ ^-^ ^27^.

A study that aimed at evaluating the health-related quality of life of cancer patients in Palliative Care and its association with sociodemographic and clinical aspects identified low schooling levels among the participants, as was the case with the findings of this research, which obtained a mean of 5.4 years of study in HERP and 7.6 at INCA^15^. These authors report that the low schooling level associated with increasing age becomes a concern with regard to these people’s ability to correctly understand diverse information, guidelines and recommendations related to health care in general^16^.

Followed by lung cancer, colorectal cancer was the most frequent in HERP. At INCA, head and neck cancer and breast cancer predominated. 

The most recent world estimate indicates that 18 million new cancer cases have occurred worldwide (17 million not counting non-melanoma skin cancer cases). Lung cancer is the most incident in the world (2.1 million) followed by breast (2.1 million), colon and rectum (1.8 million) and prostate (1.3 million). The most frequent types of cancer in men were lung (14.5%), prostate (13.5%), colon and rectum (10.9%), stomach (7.2%) and liver (6.3%). In women, the highest incidence values corresponded to breast cancer (24.2%), followed by colon and rectum (9.5%), lung (8.4%) and cervix (6.6%)^28^.

The data obtained in this study differ from those obtained with the same patient profile. In a study conducted in 2020, the authors identified that the most prevalent types of cancer in the population studied were gynecological (23.8%), gastrointestinal system (19.1%) and breast (14.3%)^20^.

In the current study, the main caregiver corresponded to a family member in both institutions. Family caregivers play an important role in the care of patients with advanced disease^29^. However, it is necessary to be aware of the emotional burden involved in the process of caring for a family member in Palliative Care, that is, it is necessary that Nursing professionals encourage family caregivers in the care duties and are aware of the identification and reduction of stressors to which they are susceptible^30^. 

In both institutions participating in the study, the number of venipunctures performed was significantly higher than the subcutaneous punctures. This fact is similar to the findings of a study that aimed at analyzing the use of hypodermoclysis in cancer patients with criteria for PC in two public general hospitals from Belo Horizonte^2^. In addition, the authors mention that there was greater use of the subcutaneous route by the service that had a Palliative Care team.

Although performance of hypodermoclysis is guided by important advantages to the intravenous route, among them greater puncture ease and lower risk of serious complications, such intervention is still underused in the clinical practice^31^. 

In HERP, the main purpose for obtaining a parenteral route was antibiotic therapy, followed by analgesia. At INCA, the main purpose was analgesia. These findings corroborate the results of another study that identified analgesia as the main purpose for parenteral access^24^. Considering that a significant range of antibiotics is incompatible with the subcutaneous route, the fact of observing a greater number of punctures in a service where the main purpose of the puncture was antibiotic therapy is plausible.

The literature points out that hospitalization of patients in Palliative Care is frequent due to the need to control unpleasant symptoms such as pain, nausea and vomiting, dyspnea and others, related to the underlying disease itself and its evolution, which exert an excessive impact on the patients’ quality of life and, therefore, need to be managed properly^24^. 

With regard to the occurrences related to hypodermoclysis, they were uncommon in the current study, manifested by local and easily resolved complications without systemic involvement. In turn, the occurrences related to venipuncture were observed in both services investigated, significantly surpassing those with subcutaneous puncture. 

A study that aimed at characterizing the complications associated with the use of the subcutaneous route in the infusion of medications and solutions in Palliative Care observed occurrence of edema and hyperemia, which are characterized by being of low severity, reversible and with little clinical repercussion for the patient. Cellulitis was also observed, although in a very small number of situations (3.5%)^32^.

On the other hand, several occurrences related to peripheral venipuncture were observed in the study participants, the most frequent being catheters with expired use periods, in addition to presenting infiltration of the medication into the subcutaneous tissue and inadequate fixation, which did not allow direct observation of the catheter insertion site. 

Complications related to the use of peripheral venous catheters are quite common^33^. A multicenter study identified that the use of peripheral venous catheters is associated with high rates of complications, such as insertion difficulties, phlebitis, infiltration and occlusion, among others, resulting in premature removal and replacement^34^. 

The findings of the current study corroborate a survey that aimed at evaluating the Nursing team regarding the punctures and maintenance of the peripheral intravenous catheters, in which several occurrences permeated maintenance of intravenous catheters. Such study identified that 53% of the participants observed presented venipunctures with blood soiling in the transparent dressing, 30.1% of the punctures were undated, consequently favoring lack of control over the adequate permanence time of the catheter^33^. The same study identified that 3% of the patients presented clinical signs such as presence of erythema, with or without local pain, being classified as Grade 1 phlebitis; 1.8% presented erythema with pain and/or edema at the catheter insertion site and were classified as Grade 2; and only 0.3% were classified as Grade 3, as they presented erythema, local pain and/or edema with hardening and palpable fibrous cord^35^. The current study indicated similar values in relation to the occurrence of phlebitis; however, only Grade 1 phlebitis occurred in both services under study.

A prospective cohort study that sought to identify inherent and modifiable risk factors related to the use of peripheral venous catheters identified catheter occlusion as the most common failure among the participants, with a rate similar to that of the current study^36^.

In view of such notes, it is observed that failures related to the use of venous catheters are common, and that the inconsistency found between what is recommended in the guidelines and the practice is real^34^. Thus, it is necessary that the supervising nurses of the inpatient units review current protocols for insertion and maintenance of peripheral intravenous catheters, paying special attention to monitoring the predisposing factors for possible complications, such as expiration date of the catheters, fixation of the dressings, protection of the catheters during the bath and choice of smaller calibers for the punctures, among others, proposed by the National Health Surveillance Agency (*Agência Nacional de Vigilância Sanitária*, ANVISA)^35^. 

In addition to constant monitoring of the parenteral therapy, nurses should evaluate the patients’ characteristics, the medications prescribed, the expected treatment length and the risk factors for the occurrence of complications. In addition to that, an assessment of the risks and benefits of each type of catheter is required, as well as considering the patients’ preferences.

Thus, considering the aspects related to the profile of cancer patients hospitalized in Palliative Care is relevant for the decision to be made in relation to opting for an intravenous or subcutaneous access. 

In this sense, the current study contributes to the advancement of scientific knowledge because it adds clear evidence on the occurrences related to venipuncture and hypodermoclysis in cancer patients, enabling the professionals to sparingly evaluate which should the most appropriate option be between both procedures in order to ensure quality care and free of harms to this profile of patients.

A limitation of this study was the impossibility of comparing the findings between both services, one being a general hospital and the other a specific service for the care of patients in Palliative Care. The number of participants in each of the services was quite discrepant, which made it impossible to carry out statistical analyses that allowed comparing the services. However, it was possible to identify diverse evidence that reinforces the current literature that hypodermoclysis presents fewer complications than peripheral venipuncture. Similar studies involving more robust methodologies and analyses may lead to the production of new evidence capable of favoring the clinical practice of hypodermoclysis. 

## Conclusion

The number of occurrences and complications related to peripheral venipuncture was considerably higher than those related to hypodermoclysis, the most common being blood soiling at the venous catheter insertion site, catheter with expired use period, infiltration and inadequate fixation, making it impossible to monitor the catheter insertion site. Phlebitis was also observed, although to a lesser extent.

Thus, it is suggested that, when compared to peripheral venipuncture, hypodermoclysis offers greater patient safety with regard to the complications resulting from punctures and maintenance of the catheters in the subcutaneous tissue.

It is recommended to train the professionals on the use of hypodermoclysis, as well as to adopt guidelines and protocols that can guide the Nursing professionals’ clinical practice in order to favor adherence to this technique. Furthermore, teaching of the hypodermoclysis practice should be encouraged in universities, in order to favor the training of professionals for its performance.

This study may contribute to changing the procedures related to the administration of medications and fluids to cancer patients in Palliative Care and, consequently, to greater adherence by Nursing professionals regarding the use of hypodermoclysis as a second route of choice, in situations of impaired oral administration. 
